# Efficacy and Safety of IncobotulinumtoxinA in the Treatment of Lower Limb Spasticity in Japanese Subjects

**DOI:** 10.3389/fneur.2022.832937

**Published:** 2022-03-17

**Authors:** Yoshihisa Masakado, Hitoshi Kagaya, Kunitsugu Kondo, Yohei Otaka, Andrzej Dekundy, Angelika Hanschmann, Thorin L. Geister, Ryuji Kaji

**Affiliations:** ^1^Department of Rehabilitation Medicine, Tokai University School of Medicine, Kanagawa, Japan; ^2^Department of Rehabilitation Medicine I, School of Medicine, Fujita Health University, Toyoake, Japan; ^3^Department of Rehabilitation Medicine, Tokyo Bay Rehabilitation Hospital, Chiba, Japan; ^4^Merz Pharmaceuticals GmbH, Frankfurt am Main, Germany; ^5^Department of Clinical Neuroscience, Tokushima University, Tokushima, Japan

**Keywords:** botulinum neurotoxin type A, incobotulinumtoxinA, treatment, spasticity, lower limb, Japan

## Abstract

**Objective:**

To confirm the efficacy and safety of incobotulinumtoxinA (Xeomin®, Merz Pharmaceuticals GmbH; total dose 400 U) in Japanese subjects with lower limb (LL) poststroke spasticity using the Modified Ashworth Scale spasticity score for the plantar flexors (MAS-PF).

**Methods:**

This phase III study (Japic clinical study database No. CTI-153030, 7 October 2015) included a double-blind, 12-week main period (MP) in which 208 subjects were randomized to receive one injection cycle of incobotulinumtoxinA 400 U *(n* = 104) or placebo (*n* = 104) in the *pes equinus* muscles, and an open-label extension (OLEX) that enrolled 202 subjects who received three injection cycles, 10–14 weeks in duration (the last cycle was fixed at 12 weeks). Changes in MAS-PF for incobotulinumtoxinA vs. placebo from baseline to Week 4 of the MP and to the end-of-cycle visits in the OLEX were evaluated.

**Results:**

The area under the curve for the change in MAS-PF was statistically significantly greater with incobotulinumtoxinA vs. placebo in the MP (mean: −7.74 vs. −4.76; least squares mean: −8.40 vs. −5.81 [*p* = 0.0041]). In the OLEX, mean changes in MAS-PF from baseline to end-of-study showed continued improvement with repeated injections. No new safety concerns were observed with the incobotulinumtoxinA treatment. Its efficacy and safety were consistent regardless of the length of the injection cycle interval in the OLEX.

**Conclusion:**

This study demonstrated that incobotulinumtoxinA (total dose 400 U) is an effective and a well-tolerated treatment for LL spasticity in Japanese subjects using flexible injection intervals of 10–14 weeks.

## Introduction

The number of individuals who survive stroke in Japan, along with the number of individuals aged over 65 years, has increased rapidly over the last 20 years (1). Around one-third of those who have a stroke develop spasticity, which often leads to pain, distress, and secondary complications (2, 3). Spasticity management is achieved through a program of patient-specific treatment goals, overseen by a multidisciplinary team of medical specialists, nurses, and therapists. Alongside physical therapy, pharmacological treatment is a key part of the treatment program (3).

Botulinum neurotoxin type A (BoNT-A) treatment is recommended for lower limb (LL) spasticity in adults (3). The BoNT-A formulation onabotulinumtoxinA has fixed labeled doses per muscle, and the dosing interval is a minimum of 12 weeks (4), reducing flexibility in dosing options. However, a survey conducted on poststroke patients in North America and Europe revealed that 43% of the patients would prefer more flexible dosing intervals for their BoNT-A injections, with reinjections after ≤ 10 weeks. This result was confirmed by physicians treating poststroke patients. Physicians also concluded that 25% of their patients could probably benefit from higher doses (5), indicating a clinical need for more individualized treatment.

IncobotulinumtoxinA (Xeomin®, Merz Pharmaceuticals GmbH) is a BoNT-A formulation (150 kD) free from complexing proteins (6). The efficacy of escalating doses of incobotulinumtoxinA (total body doses of up to 800 U) for the treatment of *pes equinovarus* and other patterns of LL poststroke spasticity has previously been demonstrated in an ancillary analysis of a phase III trial in Non-Japanese subjects (TOWER) (7). In addition, the J-PURE study showed that incobotulinumtoxinA was well tolerated at a total dose of 400 U and it decreased muscle tone and improved functionality in Japanese subjects with poststroke upper limb (UL) spasticity (8). Therefore, in June 2020, incobotulinumtoxinA was granted approval in Japan for use at a dose of up to 400 U in patients with UL spasticity (9).

Here, we present the results of the main period (MP) and open-label extension period (OLEX) of the J-PLUS study, which aimed to investigate the efficacy and safety, vs. placebo of a single incobotulinumtoxinA injection cycle (MP) and repeated incobotulinumtoxinA injections up to 52 weeks (MP and OLEX) in the treatment of poststroke LL spasticity in Japanese subjects (Japic clinical study database No. CTI-153030, first posted October 7, 2015).

## Materials and Methods

### Subjects

Male and female subjects, 20–80 years of age, and of East Asian race (recruited in Japan) were eligible for the study if they had unilateral LL spasticity with equinus foot deformity caused by a stroke at least 6 months prior to the screening visit, a bodyweight of at least 50 kg, clinical need for a total dose of incobotulinumtoxinA 400 U, a Modified Ashworth Scale (MAS) (10) spasticity sum score for the plantar flexors (MAS-PF) of ≥3 (11) at screening and the baseline injection visit, and were botulinum toxin-naïve or pretreated. The clinical need for incobotulinumtoxinA 400 U was decided according to the experience-based opinion of the investigator. This need was derived from the patient's spasticity status and the expected improvement incobotulinumtoxinA could provide. A washout period of at least 16 weeks was required between pretreatment with any BoNT for any indication and the screening visit for this study. Subjects were not eligible if they had: fixed contracture (defined as severe restriction of the range of joint movement on passive stretch) or other types of muscle hypertonia (e.g., rigidity) in the affected joint(s) intended to be treated; nonstroke-related spasticity; bilateral LL paresis, paralysis, or tetraparesis; any previous and planned surgical treatment for spasticity in the target muscles; or planned concomitant treatment with BoNT-A for any other body region during the study period.

The study was conducted in accordance with the ethical principles of the Declaration of Helsinki. Study protocols, informed consent forms, and other appropriate study-related documents were reviewed and approved by the local independent ethics committees and institutional review boards. Written informed consent was obtained from all subjects in accordance with Japanese regional laws and regulations.

### Study Design

This multicenter study enrolled subjects at Japanese sites only and consisted of three periods. The first period of the study, the lead-in tolerability period (LITP), investigated the safety and tolerability of a 400-U dose of incobotulinumtoxinA in the *pes equinus* muscles in at least 10 subjects during an observation period of 12 weeks. Based on the LITP, the dose of 400 U was deemed safe for enrollment in the second period of the study, the MP, by both the sponsor and the independent Data Monitoring Committee. In addition, this dose had proved effective and well-tolerated in the phase III TOWER study of LL poststroke spasticity in adults from the USA and Europe (7) and the phase III J-PURE study of UL poststroke spasticity in adults from Japan (8), and allowed the toe flexors to be treated if necessary, providing a more individualized treatment approach. Results of the LITP have been previously published (12) and will not be presented in this manuscript.

**Figure 1:**
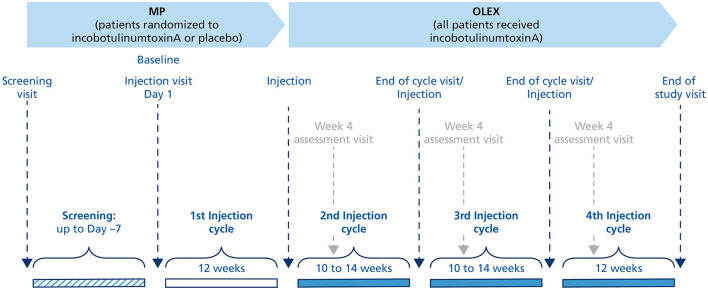
Study design. MP, main period; OLEX, open-label extension period.

The MP, the second period of the study, was designed to confirm the efficacy and safety of a single injection cycle of incobotulinumtoxinA 400 U compared with placebo in the *pes equinus* muscles during an observation period of 12 weeks (Figure 1). Subjects were randomized 1:1 to incobotulinumtoxinA or placebo (injection Cycle 1) using a computerized randomization program that assigned an individual number to each subject. Placebo vials had the same appearance as incobotulinumtoxinA vials to allow double-blinding of the subject and investigator.

The third and the final period of the study, the OLEX period, was designed to investigate the efficacy and safety of repeated incobotulinumtoxinA injections in subjects who had completed the MP or LITP and had a clinical need for *pes equinus* reinjection for a total treatment duration of up to 52 weeks. The OLEX period consisted of three incobotulinumtoxinA injection cycles, the first two (injection Cycles 2 and 3) had a flexible duration (10–14 weeks) that depended on the time-point for the need for reinjection as assessed by the investigator, but the third cycle (injection Cycle 4) was fixed at 12 weeks.

Guided injection using electromyography, nerve stimulation, or ultrasound imaging was performed at all injection sessions to identify the target muscles and facilitate injection. The injection dilution used was 50 U/mL. The total dose of incobotulinumtoxinA was fixed at 400 U in both the MP and OLEX, but there was greater flexibility in dosing in the OLEX with respect to the muscles treated and the dose range for each muscle (Supplementary Table S1). Concomitant therapies allowed during the study included oral centrally acting muscle relaxants, antidepressants, anticoagulants, physiotherapy, occupational therapy, and other rehabilitation measures to treat spasticity. Physiotherapy, occupational therapy, or any other rehabilitation measures to treat spasticity of the target limb were required to remain stable during the MP. Prohibited concomitant therapies included BoNTs other than incobotulinumtoxinA, antibiotics, or parenterally administered drugs interfering with neuromuscular transmission, phenol or alcohol injections, antispastic medications with peripheral muscle relaxants, and surgery in the target limb within 8 weeks prior to screening.

### Efficacy in LL Spasticity

The primary efficacy endpoint was the area under the curve (AUC) for the change from baseline (Day 1) in the MAS-PF throughout the MP (to Week 12). Secondary efficacy endpoints were changes in MAS-PF from baseline to Weeks 4, 6, and 8 in the MP and Week 4 and end-of-cycle visits in the OLEX, and MAS-PF response rates at Weeks 4, 6, and 8 in the MP and Week 4 and end-of-cycle visits in the OLEX (responders = subjects with a reduction of ≥1 point from baseline). Other efficacy endpoints, which were assessed at Weeks 4 and 12 in the MP and Week 4 and end-of-cycle visits in the OLEX, were changes from baseline in MAS ankle inversion/foot supination score (11), duration of response (the period between the first injection and the date at which a subject was first no longer classified as a responder), change from baseline in the investigator's clinical global impression (CGI) score (13), change from baseline in time (seconds) to walk 10 m (14), change in total gait physician's rating scale (PRS) score (11, 15), and pain in the affected ankle in the last 7 days measured using the patients' assessment of spasticity, pain, and spasms (11-point numeric rating scale). The timed 10-m walking test involved the patient walking 10 m at preferred and maximum possible speed on a measured walkway marked every 2 m. The time to walk between the 2- and 8-m markings was recorded to allow for acceleration and deceleration. One trial per speed was performed and recorded.

### Safety and Tolerability

The severity of adverse events (AEs) and their relationship to study treatment were evaluated throughout the study. AEs were coded according to the Medical Dictionary for Regulatory Activities (MedDRA) version 22.0. Serious AEs were those considered to be life-threatening, that required hospitalization, or led to death. Subjects were specifically monitored for “AEs of special interest” (AESIs), which included AEs indicative of potential toxin spread, such as difficulty in swallowing, talking or breathing, muscle weakness, and double vision. Physical and laboratory assessments were also performed at each study visit. Treatment tolerability was assessed by the investigator after each injection on a 4-point Likert scale where 1 = “very good” and 4 = “poor”.

### Neutralizing Antibodies Against BoNT-A

Blood samples were screened using a fluorescence immunoassay to detect the presence of antibodies against BoNT-A at baseline, Week 12, and at end-of-study. Samples found to be positive for antibodies against BoNT-A were analyzed using a validated mouse *ex vivo* hemidiaphragm assay (HDA).

### Statistical Analyses

Sample size was determined based on a previous study in which the treatment difference between the AUC of the change from baseline in MAS-PF up to Week 12 of BoNT-A and placebo was 3.4 (standard deviation [SD] 6.6) (11). Taking into account a target population in need of treatment with incobotulinumtoxinA 400 U, which was slightly different from the population in the study by Kaji et al. (11), the treatment difference to be detected was reduced to 3.0 for the sample size calculation. With an estimated discontinuation rate of ~6% in the MP, a sample size of 206 was determined to be adequate to provide 90% power at the 5% level of significance. There were two main analysis sets defined separately for the MP and OLEX periods: the safety evaluation set (SES; the subset of all subjects exposed to incobotulinumtoxinA at least once in the respective period) and the full analysis set (the subset of subjects in the SES with at least a baseline value for MAS-PF in the respective period).

Confirmatory analysis of the primary endpoint was performed by analysis of covariance (ANCOVA) with baseline MAS-PF as covariate and pooled site, treatment, and sex as factors. This approach was also applied to the secondary endpoint of change from baseline in MAS-PF to Weeks 4, 6, and 8 of the MP. For the primary endpoint, in case of isolated missing values, the AUC was calculated by using the subject's nonmissing values. The remaining missing values were imputed from baseline MAS-PF (baseline observation carried forward; BOCF). This was considered a conservative approach. The BOCF approach was also applied to the secondary endpoint of change from baseline in MAS-PF.

Comparisons between treatment groups for response rates in MAS-PF were analyzed by a logistic regression model, with response to treatment as the dependent variable, baseline MAS-PF as covariate and pooled site, treatment, and sex as factors. Missing values were imputed using a worst-case approach, where subjects with missing data would be treated as nonresponders. ANCOVA models were applied to the CGI score, and change in time to walk 10 m, change from baseline in MAS ankle inversion/foot supination score, changes in PRS by gait parameter, and change in ankle pain score with baseline as covariate and pooled site, treatment, and sex as factors, similar to the primary and secondary efficacy analysis. The logistic regression model was applied to change from baseline in MAS ankle inversion/foot supination score. Safety endpoints were analyzed using descriptive statistics and were summarized based on treatment and total population.

An analysis was also performed in which summary statistics for changes in the MAS-PF from baseline (Day 1) to the Week 4 visit following each injection in the OLEX were generated according to the length of injection Cycles 2 and 3 (10 weeks, >10–12 weeks, >12–14 weeks). A similar descriptive analysis was performed for AEs by means of frequency tables. These analyses were performed only in patients with the same injection cycle length for the OLEX injection Cycles 2 and 3.

## Results

### Subjects

**Figure 2:**
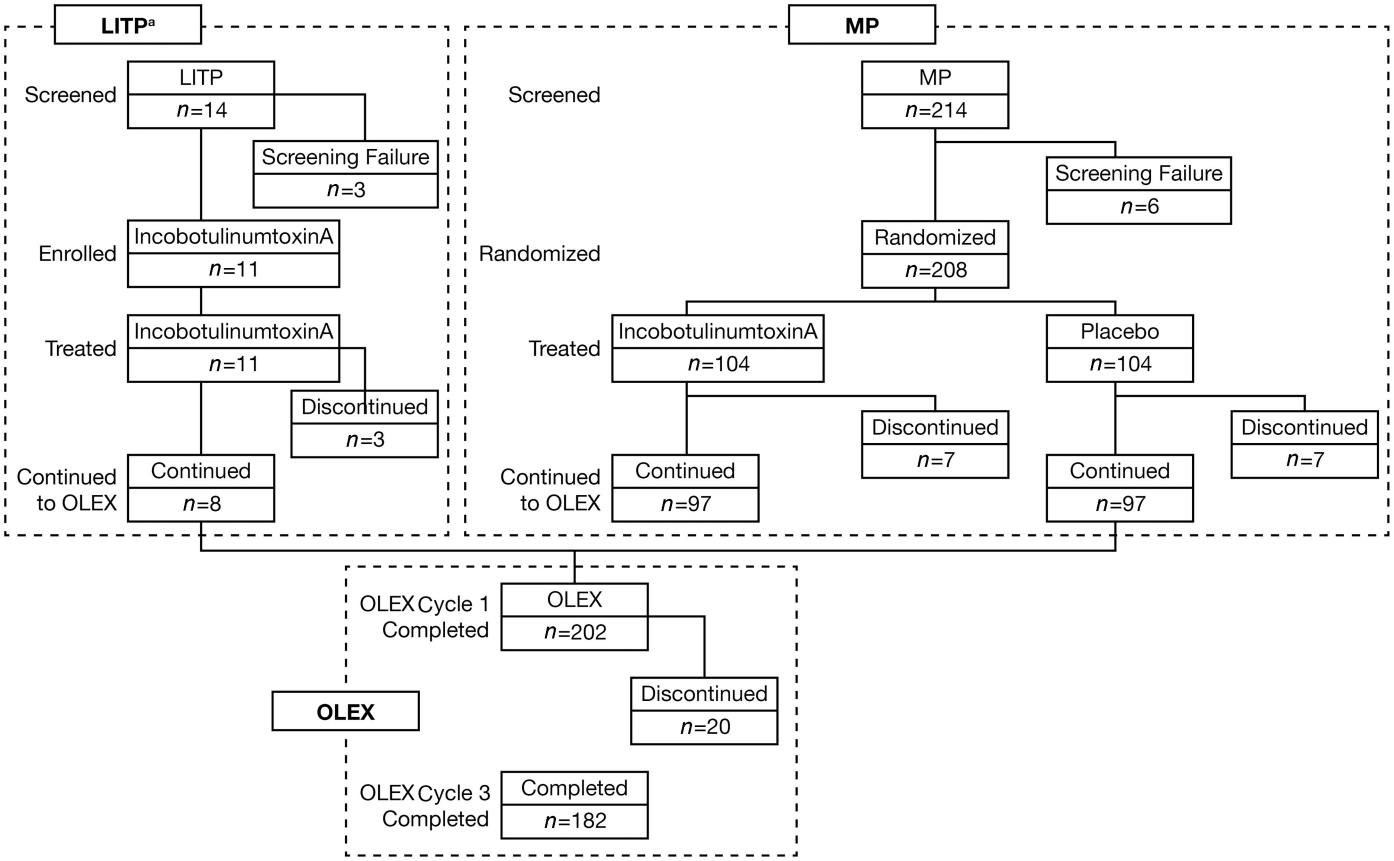
Subject disposition. ^a^The LITP was performed before the MP. LITP, lead-in tolerability period; MP, main period; *n*, number of subjects at each stage; OLEX, open-label extension period.

Beginning in November 2015, 11 Japanese subjects (8 men and 3 women) were enrolled in the LITP, 8 of whom entered the OLEX (Figure 2). In the MP (start date November 2016), 208 Japanese subjects were enrolled across 48 sites, 14 of whom discontinued. Reasons for discontinuation of incobotulinumtoxinA and placebo, respectively, were AEs (1 and 2 patients), patient withdrawal (4 and 3 patients), physician decision (1 and 1 patient), and others (1 and 1 patient). Around three-quarters of subjects enrolled in the MP were men, and the mean weight of subjects was 66.8 kg. Demographics and clinical characteristics were similar in both treatment arms. A total of 202 subjects entered the OLEX and 20 discontinued. Reasons for discontinuation were AEs (4 patients), patient withdrawal (5 patients), physician decision (2 patients), and others (9 patients). Over three-quarters of subjects in the OLEX were men and the mean weight of subjects was 66.7 kg. Around half the subjects in the MP (52.4%) and OLEX (53.0%) had been pretreated with BoNT-A (Table 1). In the OLEX period, 57.9% of subjects (117/202) had an injection interval of 10 weeks for injection Cycles 2 and 3. A total of 137 patients received OLEX injection Cycles 2 and 3 at the same time interval and provided data for the analyses of change in MAS-PF and the incidence of AEs according to injection cycle length.

**Table 1:** Subject demographics and clinical characteristics (SES).

	**MP**			**OLEX**
	**IncobotulinumtoxinA** **(*N* = 104)**	**Placebo** **(*N* = 104)**	**Total** **(*N* = 208)**	**Total** **(*N* = 202)**
Sex, *n* (%)				
Male	74 (71.2)	84 (80.8)	158 (76.0)	155 (76.7)
Age (years), mean (SD)	59.5 (11.2)	58.8 (11.0)	59.2 (11.1)	58.7 (11.1)
Height (cm), mean (SD)	164.0 (8.8)	165.4 (8.1)	164.7 (8.5)	164.9 (8.4)
Weight (kg), mean (SD)	65.9 (9.5)	67.6 (12.0)	66.8 (10.8)	66.7 (10.5)
Type of stroke, *n* (%)				
Ischemic	30 (28.8)	33 (31.7)	63 (30.3)	58 (28.7)
Hemorrhagic	74 (71.2)	71 (68.3)	145 (69.7)	144 (71.3)
Clinical pattern of LL spasticity, *n* (%)^*^				
Flexed hip	4 (3.8)	6 (5.8)	10 (4.8)	10 (5.0)
Adducted thighs	12 (11.5)	11 (10.6)	23 (11.1)	24 (11.9)
Flexed knee	89 (85.6)	84 (80.8)	173 (83.2)	166 (82.2)
Stiff knee	17 (16.3)	19 (18.3)	36 (17.3)	34 (16.8)
Equinovarus foot	104 (100.0)	104 (100.0)	208 (100.0)	202 (100.0)
Great toe extension	13 (12.5)	7 (6.7)	20 (9.6)	20 (9.9)
Time since first diagnosis of LL spasticity (months), mean (SD)	68.3 (62.9)	70.3 (63.1)	69.3 (62.8)	66.4 (59.5)
Time since the last stroke leading to spasticity (months), mean (SD)	79.8 (65.0)	86.0 (69.5)	82.9 (67.2)	79.4 (64.6)
MAS-PF baseline score, mean (SD)	3.0 (0.0)	3.0 (0.0)	3.0 (0.0)	3.0 (0.0)
Ankle pain score, mean (SD)^§^	1.9 (2.7)	2.3 (3.0)	2.1 (2.8)	1.7 (2.5)
Pre-treatment with BoNT-A, *n* (%)	52 (50.0)	57 (54.8)	109 (52.4)	107 (53.0)
Physical therapies, *n* (%)^#^				
Physiotherapy	60 (57.7)	54 (51.9)	114 (54.8)	107 (53.0)
Occupational therapy	28 (26.9)	27 (26.0)	55 (26.4)	57 (28.2)
Rehabilitation therapy	4 (3.8)	12 (11.5)	16 (7.7)	18 (8.9)
Use of mobility aids, *n* (%)				
Orthosis	69 (66.3)	69 (66.3)	138 (66.3)	138 (68.3)
Walking aid	18 (27.3)	9 (8.7)	27 (13.0)	25 (12.4)
Wheelchair	3 (2.9)	5 (4.8)	8 (3.8)	8 (4.0)

* *Multiple entries possible*.

§ *Item 2 of the Patient's Assessment of Spasticity, Pain, and Spasms scale, which rates the severity of pain on an 11-point numeric rating scale from 0 (no pain) to 10 (worst possible pain)*.

# *Fewer than 3% of the total MP population received kinesitherapy (2.9%), massage (2.9%), muscle electrostimulation therapy (1.4%), acupuncture (1.0%), fecal disimpaction (0.5%), or heat therapy (0.5%). All physical therapies remained stable throughout the MP*.

*BoNT-A, botulinum neurotoxin type A; LL, lower limb; MP, main period; N, number of subjects in each period; n, number of subjects in the given category; OLEX, open-label extension period; SD, standard deviation; SES, safety evaluation set*.

### Efficacy in LL Spasticity

**Figure 3:**
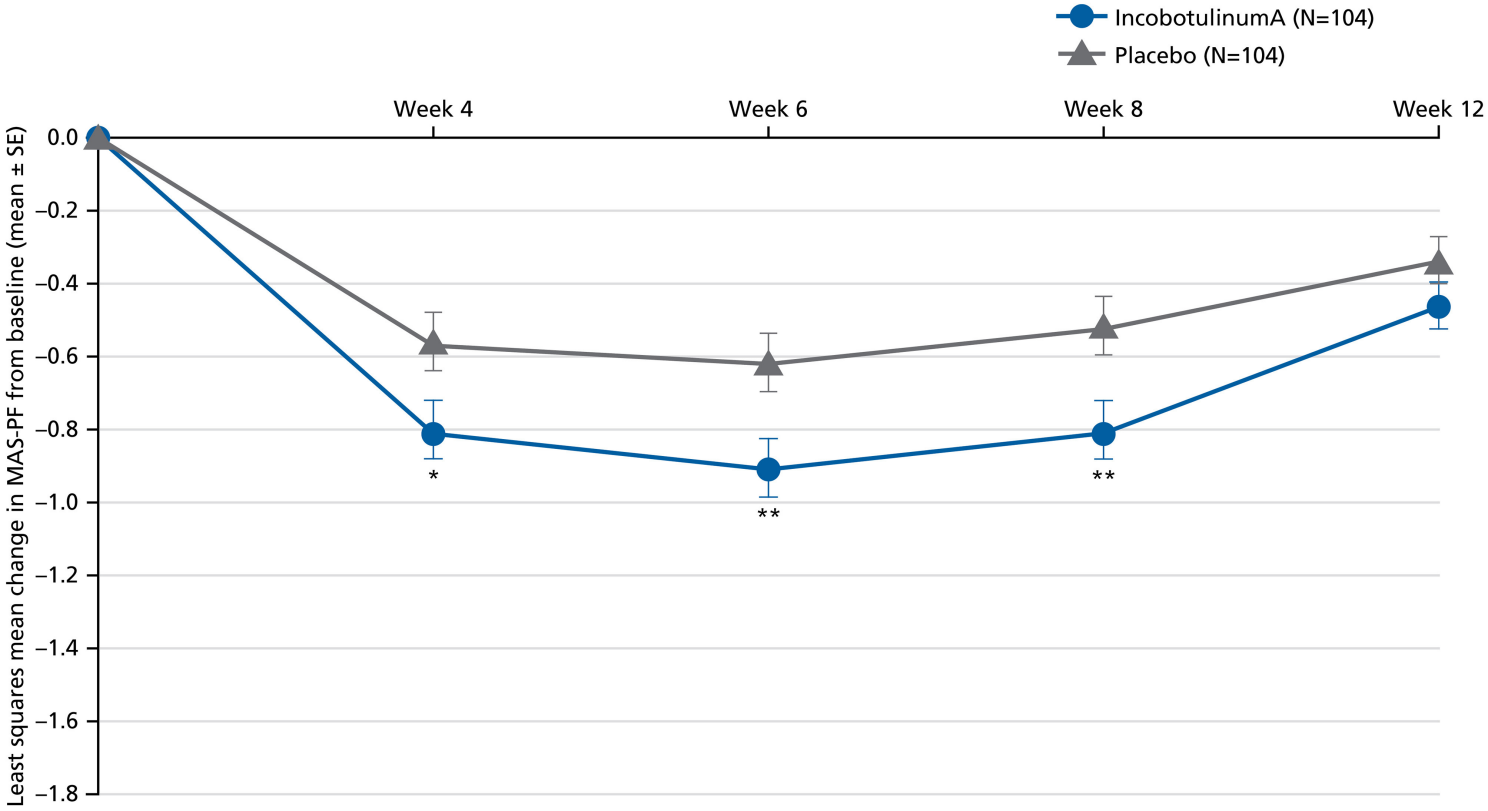
Least squares mean change in MAS-PF from baseline to Weeks 4, 6, 8, and 12 in the MP (BOCF, FAS; ANCOVA). ANCOVA, analysis of covariance; BOCF, baseline observation carried forward; FAS, full analysis set; MAS-PF, Modified Ashworth Scale spasticity score for the plantar flexors, MP, main period; SE, standard error. *p < 0.05. **p < 0.01 (vs. placebo). Error bars represent the SE.

The primary endpoint, AUC for the change in the MAS-PF from baseline to Week 12 of the MP, demonstrated statistically significantly greater LL spasticity improvements in the incobotulinumtoxinA group vs. the placebo group (Table 2 and Figure 3) regardless of baseline MAS-PF score, sex, or pooled site.

**Table 2:** Summary statistics and ANCOVA of AUC for the change from baseline in MAS-PF to the end of MP (BOCF, FAS).

	**IncobotulinumtoxinA** **(*N* = 104)**	**Placebo** **(*N* = 104)**
Mean AUC ± SD	−7.74 ± 7.01	−4.76 ± 5.84
LS mean AUC ± SE	−8.40 ± 0.661	−5.81 ± 0.713
LS mean AUC difference (incobotulinumtoxinA–placebo) ± SE	−2.59 ± 0.892	
95% CI	−4.35; −0.83	
*p*-value	0.0041	

*ANCOVA, analysis of covariance; AUC, area under the curve; BOCF, baseline observation carried forward; CI, confidence interval; FAS, full analysis set; LS, least squares; MAS-PF, Modified Ashworth Scale spasticity score for the plantar flexors; MP, main period; N, number of subjects in each group; SD, standard deviation; SE, standard error*.

Improvements were also generally seen in secondary endpoints in subjects receiving incobotulinumtoxinA in the MP and OLEX. Improvements with incobotulinumtoxinA were statistically significantly greater than placebo at each time-point for changes from baseline in MAS-PF at Weeks 4, 6, and 8, with the largest effect at Week 6 (Figure 3). In the OLEX, mean (SD) changes in MAS-PF from study baseline to the control visits 4 weeks after each injection in Cycles 2, 3, and 4 were −1.05 (0.75), −1.16 (0.77), and −1.18 (0.73), respectively (Supplementary Figure S1), and −0.51 (0.63), −0.60 (0.65), and −0.83 (0.77), respectively, at the end of Cycles 2 and 3 and end-of-study, indicating further spasticity improvements across repeated injection cycles.

**Figure 4:**
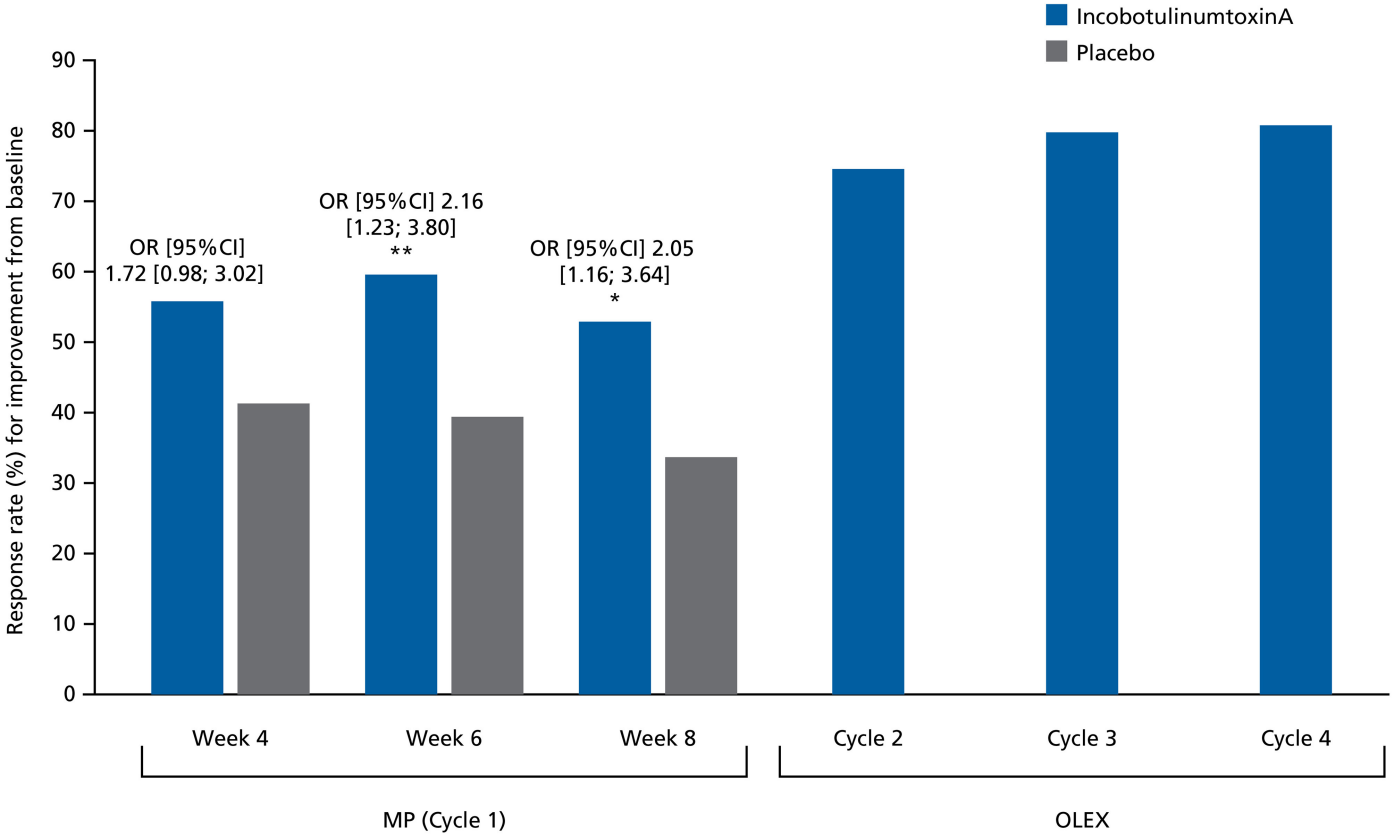
Response rates (%) for at least 1-point improvement from baseline in MAS-PF at Weeks 4, 6, and 8 in the MP and at the Week 4 assessment visit during Cycles 2, 3, and 4 in the OLEX (worst-case approach, FAS). Comparisons between treatment groups were performed using a logistic regression model where response to treatment was the dependent variable, baseline MAS-PF score was a covariate, and site (pooled), treatment and sex were factors. CI, confidence interval; FAS, full analysis set; MAS-PF, Modified Ashworth Scale spasticity score for the plantar flexors; MP, main period; OLEX, open-label extension period; OR, odds ratio. *p < 0.05. **p < 0.01.

The MAS-PF response rates in the MP were consistently higher in the incobotulinumtoxinA group vs. placebo at Weeks 4, 6, and 8 (Figure 4), with the difference reaching statistical significance at Weeks 6 and 8. The odds of being a responder were at least 1.5-fold higher in the incobotulinumtoxinA group vs. the placebo group at all assessment times during the MP. Response rates from study baseline to Week 4 of Cycles 2, 3, and 4 in the OLEX were 150/201 (74.6%), 150/188 (79.8%), and 147/182 (80.8%), respectively, and to end of the OLEX was 108/182 (59.3%), indicating durable results across repeated injection cycles.

In the analysis of change in MAS-PF from baseline according to the length of injection Cycles 2 and 3 in the OLEX, patients achieved similar benefits irrespective of cycle length (Supplementary Figure S2).

Similar to the change from baseline in MAS-PF, least squares mean (standard error) change from baseline in MAS ankle inversion/foot supination score in the MP was also significantly greater in the incobotulinumtoxinA group than the placebo group at Week 4 (−0.7 [0.1] vs. −0.5 [0.1]; *p* = 0.016), 6 (−0.8 [0.1] vs. −0.5 [0.1]; *p* = 0.0003), and 8 (−0.6 [0.1] vs. −0.4 [0.1]; *p* = 0.0034), with the largest effect seen at Week 6.

Furthermore, the duration of response for 1-point responders was longer in subjects who received incobotulinumtoxinA compared with those who received placebo in plantar flexors (*p* = 0.1055) and ankle inversion/foot supination (*p* = 0.1410) in the MP. The ≥1-point response for plantar flexors and ankle inversion/foot supination persisted for more than 10 weeks after each injection cycle in the OLEX period, slightly increasing at each injection cycle.

No notable differences in response profiles between BoNT-A pretreated and treatment-naïve subjects were observed in the MP.

For the investigator's CGI, 10-m walking time, total gait PRS scores, and ankle pain scores slight numerical (but not statistically significant) differences in favor of the incobotulinumtoxinA group were observed in the MP, and these slight improvements were maintained in the OLEX (Supplementary Table S2 and Supplementary Figures S3, S4). These results indicate a sustained improvement in LL spasticity outcomes following administration of incobotulinumtoxinA.

### Safety and Tolerability

Investigator's global assessment of tolerability was “very good” or “good” in 97/104 (93.3%) and “moderate” in 6/104 (5.8%) subjects receiving incobotulinumtoxinA in the MP. In subjects receiving placebo, tolerability was “very good” or “good” in 94/104 (90.4%), “moderate” in 7/104 (6.7%), and “poor” in 2/104 (1.9%) subjects. Data for one subject were missing in each group.

AEs were observed in almost half the subjects in both treatment arms in the MP (Table 3). Treatment-related AEs were reported in six (5.8%) and five (4.8%) subjects receiving incobotulinumtoxinA and placebo, respectively. One treatment-related serious AE (severe cellulitis, resolved) was reported in a subject receiving incobotulinumtoxinA. The remaining treatment-related AEs were nonserious and mild or moderate in severity. Treatment-related AESIs occurred in three (2.9%) and two (1.9%) subjects receiving incobotulinumtoxinA and placebo, respectively. The most common treatment-related AESI was muscular weakness (two patients [1.9%] in the incobotulinumtoxinA group and two patients [1.9%] in the placebo group).

**Table 3:** Treatment-related AEs reported in MP and OLEX by preferred term (SES).

	**MP**		**OLEX (3 injection cycles)**			
	**IncobotulinumtoxinA** **(*N* = 104)**	**Placebo** **(*N* = 104)**	**Cycle 2** **(*N* = 202)**	**Cycle 3** **(*N* = 190)**	**Cycle 4** **(*N* = 184)**	**Total** **(*N* = 202)**
Subjects with at least one AE, n (%)	50 (48.1)	51 (49.0)	81 (40.1)	66 (34.7)	63 (34.2)	131 (64.9)
Treatment-related AEs						
Subjects with at least one treatment-related AE, *n* (%)	6 (5.8)	5 (4.8)	4 (2.0)	4 (2.1)	8 (4.3)	14 (6.9)
Muscular weakness	2 (1.9)	2 (1.9)	1 (0.5)	0	3 (1.6)	3 (1.5)
Myalgia	0	3 (2.9)	1 (0.5)	0	0	1 (0.5)
Fall	0	1 (1.0)	2 (1.0)	0	0	2 (1.0)
Constipation	1 (1.0)	0	1 (0.5)	1 (0.5)	0	2 (1.0)
Limb discomfort	0	0	0	1 (0.5)	1 (0.5)	2 (1.0)
Blood creatine phosphokinase increased	0	0	0	1 (0.5)	1 (0.5)	2 (1.0)
Pain in extremity	0	1 (1.0)	0	0	1 (0.5)	1 (0.5)
Ligament sprain	0	0	0	1 (0.5)	0	1 (0.5)
Arthralgia	0	0	0	0	1 (0.5)	1 (0.5)
Malaise	1 (1.0)	0	0	0	0	0
Cellulitis	1 (1.0)	0	0	0	0	0
Post-micturition dribble	1 (1.0)	0	0	0	0	0
Incontinence	0	1 (1.0)	0	0	0	0
Neurogenic bladder	0	1 (1.0)	0	0	0	0
Contusion	0	1 (1.0)	0	0	0	0
Dizziness	0	1 (1.0)	0	0	0	0
Pollakiuria	0	0	0	0	1 (0.5)	1 (0.5)
Urinary retention	0	0	0	0	1 (0.5)	1 (0.5)
Hemorrhage subcutaneous	0	0	0	1 (0.5)	0	1 (0.5)
Hyperkeratosis	0	0	1 (0.5)	0	0	1 (0.5)
Paralysis	0	0	1 (0.5)	0	0	1 (0.5)
Treatment-related AESIs						
Subjects with at least one treatment-related AESI, *n* (%)	3 (2.9%)	2 (1.9)	3 (1.5)	1 (0.5)	4 (2.2)	7 (3.5)
Muscular weakness	2 (1.9)	2 (1.9)	1 (0.5)	0	3 (1.6)	3 (1.5)
Constipation	1 (1.0)	0	1 (0.5)	1 (0.5)	0	2 (1.0)
Urinary retention	0	0	0	0	1 (0.5)	1 (0.5)
Paralysis	0	0	1 (0.5)	0	0	1 (0.5)

*AE, adverse event; AESI, adverse event of special interest; MP, main period; N, number of subjects in each group; n, number of subjects in the given category; OLEX, open-label extension period; SES, safety evaluation set*.

AEs were observed in 131/202 (64.9%) subjects across the three OLEX cycles (Table 3). The number of AEs reported decreased slightly with each cycle. Treatment-related AEs were reported in 14/202 (6.9%) subjects during the OLEX, no treatment-related serious AEs were reported, and all treatment-related AEs were mild or moderate in severity. No subject reported generalized weakness during the study and no deaths occurred. Treatment-related AESIs occurred in seven subjects (3.5%) in the OLEX. As in the MP, the most common treatment-related AESI was muscular weakness (three subjects, 1.5%). In the OLEX, the frequency of treatment-related AEs and AESIs appeared to be consistent regardless of the length of injection cycles 2 and 3 (Supplementary Table S3).

### Neutralizing Antibodies Against BoNT-A

Using the HDA method, one subject in the incobotulinumtoxinA group and one subject in the placebo group were positive for neutralizing anti-BoNT-A antibodies at the end-of-cycle visit of the MP and at the end-of-study visit. Both subjects had received pretreatment with BoNT-As for their spasticity (not incobotulinumtoxinA as this was not on the Japanese market at the time). Most importantly, both subjects were also positive for neutralizing antibodies at baseline. There was no indication of secondary nonresponse in these subjects at the end of the study.

## Discussion

This phase III study demonstrated the efficacy and safety of incobotulinumtoxinA at a dose of 400 U in a Japanese population with LL spasticity. Notably, demographics were typical of Japanese subjects with poststroke spasticity (8). Improvements in MAS-PF and significant differences vs. placebo in MAS-PF response rates were observed in the MP. The improvements in MAS-PF were sustained across repeated incobotulinumtoxinA injection cycles in the OLEX period. MAS-PF did not fully return to baseline levels at any time during the study, and continuous numerical improvement in muscle tone was observed over time, which suggests that repeated injection cycles of incobotulinumtoxinA in subjects with LL spasticity may provide continued benefit to many patients. This is further supported by small, continued improvements from baseline in investigators CGI score, time to walk 10 m, duration of response, MAS ankle inversion/foot supination score, total gait PRS score, and ankle pain score. IncobotulinumtoxinA was well tolerated with no safety concerns observed.

This is the first study showing significant improvements in LL spasticity in subjects treated with incobotulinumtoxinA at a total dose of 400 U in a Japanese population, following the J-PURE study for UL spasticity (8). Similar to the J-PURE study (8), a statistically significant difference between incobotulinumtoxinA and placebo was observed in the primary endpoint, which was AUC of the change from baseline in MAS score for the target clinical pattern. AUC of the change from baseline in MAS-PF was selected for this study because it was considered a more accurate and sensitive outcome that better reflects the integrated changes over the entire observational period of the MP compared with assessing outcomes at a specific time-point due to differences in individual peak efficacy (11, 16, 17). Most importantly, changes from baseline in MAS-PF scores supported the efficacy of incobotulinumtoxinA, with improvements at Weeks 4, 6, and 8, and peak improvement at Week 6. Similar results were observed in a study of onabotulinumtoxinA in the treatment of LL spasticity (11).

In the current study, investigators gave numerically higher CGI scores to subjects who received incobotulinumtoxinA than to those administered placebo; these outcomes were similar to the significant improvements in CGI seen with incobotulinumtoxinA in the J-PURE study (8). Improvements in CGI were also observed with onabotulinumtoxinA in the treatment of LL spasticity (11, 18). Numerical improvements in the time to walk 10 m at preferred speed and PRS were also observed with incobotulinumtoxinA in the current study, and similar improvements were observed in a study of onabotulinumtoxinA in LL spasticity (11).

Due to differences in efficacy endpoints, it is difficult to compare the efficacy results of the current study with the TOWER study of incobotulinumtoxinA in Non-Japanese subjects with UL and LL spasticity. However, the results were generally similar with improved efficacy and a comparable safety profile being observed in subjects who received incobotulinumtoxinA (19). Overall, the results presented in this study are similar to previous studies of BoNT-A in Japanese and Non-Japanese subjects with UL and LL spasticity (8, 11, 18–20).

IncobotulinumtoxinA 400 U was well tolerated in the LITP of J-PLUS (12), and the incidence of AEs in the MP and the OLEX (48 and 34–40%, respectively) was lower than that in the LITP (73%). The incidence of AEs was also similar to that observed in a study in Japanese subjects with poststroke LL spasticity treated with 300 U onabotulinumtoxinA, the J-PURE study of incobotulinumtoxinA in Japanese subjects with poststroke UL spasticity, and the TOWER study in Non-Japanese subjects with combined UL and LL spasticity (8, 11, 19), and slightly lower than that observed in a multinational phase III study of onabotulinumtoxinA in subjects with poststroke LL spasticity (18).

In this study, two subjects were positive for neutralizing antibodies at the end-of-study visit; both subjects had a pretreatment history with other BoNTs. Therefore, there were no cases of neutralizing antibody formation in toxin-naïve subjects during this study. Similar to the J-PURE and TOWER studies (8, 19), there was no indication of the development of secondary nonresponse in subjects who were positive for neutralizing antibodies at the end-of-study visit.

In the current analyses, the efficacy and safety of incobotulinumtoxinA were shown to be consistent regardless of injection cycle interval (10 weeks vs. >10–12 and >12–14 weeks). As highlighted in the Introduction, a survey of poststroke patients in North America and Europe (5) showed that there is a clinical need for greater flexibility of dosing with BoNT-A. The results of our study suggest that incobotulinumtoxinA injection cycles can be flexible, with repeat dosing anywhere between 10 and 14 weeks depending on individual clinical needs. Thus, the treatment of LL spasticity with incobotulinumtoxinA using flexible intervals based on clinical needs can be considered effective and well tolerated in Japanese subjects.

There were a few limitations in this study. First, placebo response may be considered relatively high. However, high placebo response rates are commonly observed in spasticity studies using assessments such as the MAS (11, 18, 21–23). Several factors may contribute to this high placebo response rate, including the difficulty in assessing spasticity in the ankle joint using the MAS, as the range of motion in the ankle is narrower and thus more difficult to assess than in other joints (e.g., the elbow). In our study, we made every attempt to standardize MAS ratings through a clear and detailed study protocol, investigator training and meetings, newsletters, and monitoring. Second, changes in the assessed parameters may have been influenced by individual poststroke history and patient functioning. The measured parameters may improve stepwise based on individual status, but this could not be captured as patient-centered goal setting was not integrated into the study. It would be interesting to explore this in future studies. Third, results from the injection cycle length analyses should be viewed with caution as patient numbers for the two longer injection interval categories were low. However, the study population was large compared with similar studies (8, 11, 18), and the study design allowed subjects to benefit from further treatment in the OLEX. Finally, hemorrhagic strokes are more common in the Asian populations than in other ethnic and Caucasian populations (24, 25). People who experience a hemorrhagic stroke are at greater risk of developing poststroke spasticity than those who experience an ischemic stroke (26). Therefore, it is possible that the results from our study may vary according to the patient's background. Again, it would be interesting to explore this in future analyses.

The present study in a Japanese population confirmed the efficacy and safety of incobotulinumtoxinA for the treatment of LL spasticity, which was generally in agreement with TOWER, a previous open-label study enrolling Non-Japanese subjects (19). The findings in this study were also similar to those observed in Japanese subjects with UL spasticity (8). Furthermore, the safety profile of incobotulinumtoxinA in the MP and the OLEX of this study was similar to that observed in the LITP period (12).

Results from the MP and OLEX periods of the phase III J-PLUS study show that incobotulinumtoxinA is effective in the treatment of LL spasticity in Japanese subjects at a total dose of 400 U and has a favorable safety and tolerability profile in this population. The results also support the efficacy and safety of repeated incobotulinumtoxinA injections, with flexible dosing intervals based on clinical needs. IncobotulinumtoxinA 400 U represents an effective and well-tolerated treatment option for LL spasticity in Japanese subjects.

## Data Availability Statement

The datasets presented in this study can be found in online repositories. The name of the repository and accession number can be found below: The Japan Pharmaceutical Information Center (JAPIC) Clinical Study Database, https://rctportal.niph.go.jp/en, CTI-153030.

## Ethics Statement

The study was conducted in accordance with the ethical principles of the Declaration of Helsinki. Study protocols, informed consent forms, and other appropriate study-related documents were reviewed and approved by the local independent Ethics Committees and Institutional Review Boards (detailed in Supplementary Material). The patients/participants provided their written informed consent to participate in this study.

## Author Contributions

AD and AH contributed to the study conception and design. TG created the questionnaires used in the study. Material preparation, data collection, and analysis were performed by AD and AH. YM, HK, KK, YO, and RK contributed to acquisition and interpretation of data for the study. All authors critically revised the manuscript and approved the final manuscript for submission.

## Funding

This study was funded by Merz Pharmaceuticals GmbH in accordance with Good Publication Practice (GPP3) guidelines.

## Conflict of Interest

This study received funding from Merz Pharmaceuticals GmbH in accordance with Good Publication Practice (GPP3) guidelines. The funder had the following involvement with the study: study design; data collection, analysis, and interpretation; the writing of this article; and the decision to submit the article for publication. AD, TG, and AH are employees of Merz Pharmaceuticals GmbH. RK is a recipient of consultancy fees from Merz Pharmaceuticals GmbH. The remaining authors declare that the research was conducted in the absence of any commercial or financial relationships that could be construed as a potential conflict of interest.

## Publisher's Note

All claims expressed in this article are solely those of the authors and do not necessarily represent those of their affiliated organizations, or those of the publisher, the editors and the reviewers. Any product that may be evaluated in this article, or claim that may be made by its manufacturer, is not guaranteed or endorsed by the publisher.
