# Supplementary material for: Efficacy and Safety of IncobotulinumtoxinA in the Treatment of Lower Limb Spasticity in Japanese Subjects
Source: Front Neurol. 2022 Mar 17;13:832937. doi: 10.3389/fneur.2022.832937 (PMC8970182; doi:10.3389/fneur.2022.832937)
Supplement: Supplementary file 1 [file Data_Sheet_1.docx]

**Table S1.** Injection schemes for the treatment of lower limb spasticity with incobotulinumtoxinA 400 U in the MP and OLEX with and without the need to treat toe muscles

| **Target muscle** | **Units of incobotulinumtoxinA** | **Volume injected (mL)**^a^ | **Number of injection sites**^a^ |
| --- | --- | --- | --- |
| **MP with need to treat toe muscles** | | | |
| Gastrocnemius (medial and lateral head)^b^ | 75 + 75 | 1.5 + 1.5 | 4–6 |
| Soleus^b^ | 75 | 1.5 | 2–4 |
| Tibialis posterior^b^ | 75 | 1.5 | 2–3 |
| Flexor digitorum longus^b^ | 50 | 1.0 | 1–3 |
| Flexor hallucis longus^b^ | 50 | 1.0 | 1–2 |
| **MP without need to treat toe muscles** | | | |
| Gastrocnemius (medial and lateral head)^b^ | 100 + 100 | 2.0 + 2.0 | 4–6 |
| Soleus^b^ | 100 | 2.0 | 2–4 |
| Tibialis posterior^b^ | 100 | 2.0 | 2–3 |
| **OLEX with need to treat toe muscles** | | | |
| Gastrocnemius (medial and lateral head)^b^ | 50–200 | 1.0–4.0 | 2–6 |
| Soleus^c^ | 50–200^d^ | 1.0–4.0^d^ | 2–4 |
| Tibialis posterior | 50–150^d^ | 1.0–3.0^d^ | 2–3 |
| Flexor digitorum longus | 50–100^d^ | 1.0–2.0^d^ | 1–3 |
| Flexor hallucis longus | 25–75^d^ | 0.5–1.5^d^ | 1–2 |
| **OLEX without need to treat toe muscles** | | | |
| Gastrocnemius (medial and lateral head)^b^ | 50–200 | 1.0–4.0 | 2–6 |
| Soleus^c^ | 50–200^d^ | 1.0–4.0^d^ | 2–4 |
| Tibialis posterior | 50–150^d^ | 1.0–3.0^d^ | 2–3 |

^a^Maximum 1.0 mL per injection site.

^b^Injection of these muscles was mandatory.

^c^Injection into this muscle was advised.

^d^If the muscle was chosen for injection.

MP, main period; OLEX, open-label extension period.

**Table S2.** Mean change from baseline in other efficacy endpoints in adults with lower limb spasticity treated with incobotulinumtoxinA 400 U in the MP (least squares mean change with SE; FAS) and OLEX (mean change with SD; FAS).

| **Timepoint** | **Investigator’s CGI** | | | **10-meter walking time (seconds)** | | | | | | **Total gait PRS score** | | **Ankle pain score^a^** | | |
| --- | --- | --- | --- | --- | --- | --- | --- | --- | --- | --- | --- | --- | --- | --- |
|  |  |  |  | **At preferred speed** | | | **At maximum speed** | | |  |  |  |  |  |
|  | **n/N** | **Change** | **n/N** | | **Change** | **n/N** | | **Change** | **n/N** | | **Change** | | **n/N** | **Change** |
| **MP (LS mean change from baseline with SE; ANCOVA, BOCF)** | | | | | | | | | | | | | | |
| **Week 4** |  |  |  | |  |  | |  |  | |  | |  |  |
| IncobotulinumtoxinA | 104/104 | 1.4 (0.2) | – | | – | – | | – | 58/104 | | 0.6 (0.2) | | 104/104 | -0.6 (0.2) |
| Placebo | 104/104 | 1.4 (0.2) | – | | – | – | | – | 64/104 | | 0.5 (0.2) | | 104/104 | -0.7 (0.2) |
| **Week 12** |  |  |  | |  |  | |  |  | |  | |  |  |
| IncobotulinumtoxinA | 104/104 | 0.8 (0.2) | 56/104 | | -1.0 (1.0) | 56/104 | | -1.2 (1.4) | 58/104 | | 0.1 (0.2)* | | 104/104 | -0.6 (0.2) |
| Placebo | 104/104 | 1.0 (0.2) | 60/104 | | -0.5 (1.0) | 60/104 | | 0.7 (1.4) | 64/104 | | 0.6 (0.2) | | 104/104 | -0.5 (0.2) |
| **OLEX (mean change from baseline with SD)** | | | | | | | | | | | | | | |
| Cycle 2 (Week 4) | 194/201 | 2.4 (2.1) | 106/202 | | -0.4 (4.3)^b^ | 106/202 | | -0.4 (4.1)^b^ | 111/201 | | 1.1 (1.6) | | 194/201 | -1.0 (2.7) |
| Cycle 3 (Week 4) | 182/188 | 2.4 (2.2) | – | | – | – | | – | 103/188 | | 1.1 (1.6) | | 182/188 | -1.1 (2.7) |
| Cycle 4 (Week 4) | 176/182 | 2.4 (2.2) | – | | – | – | | – | 98/182 | | 1.1 (1.7) | | 176/182 | -1.1 (2.7) |
| End of study (Cycle 4, Week 12) | 176/182 | 2.0 (2.2) | 93/182 | | -0.1 (10.7) | 93/182 | | -0.2 (10.2) | 98/182 | | 0.8 (1.7) | | 176/182 | -1.0 (2.7) |

^a^Item 2 of the Patient’s Assessment of Spasticity, Pain and Spasms scale.

^b^Day 1 of cycle 2.

*p<0.05 vs. placebo.

ANCOVA, analysis of covariance; BOCF, baseline observation carried forward; CGI, clinical global impression; FAS, full analysis set; LS, least squares; MP, main period; n, number of non-missing observations; N, total number of subjects; OLEX, open-label extension period; PRS, physician’s rating scale; SD, standard deviation; SE, standard error.

**Table S3** AEs reported in the OLEX according to the length of injection cycles 2 and 3 (SES)

|  | **Length of injection Cycles 2 and 3** | | |
| --- | --- | --- | --- |
|  | **10 weeks**  **(N = 117)** | **>10–12 weeks**  **(N = 14)** | **>12–14 weeks**  **(N = 6)** |
| Subjects with at least one AE, n (%) | 74 (63.2) | 12 (85.7) | 6 (100.0) |
| Serious AEs | 5 (4.3) | 1 (7.1) | 1 (16.7) |
| Treatment-related AEs | | | |
| Subjects with at least one treatment-related AE, n (%) | 5 (4.3) | 3 (21.4) | 1 (16.7) |
| Serious treatment-related AEs | 0 | 0 | 0 |
| Pollakiuria | 1 (0.9) | 0 | 0 |
| Urinary retention | 1 (0.9) | 0 | 0 |
| Blood creatine phosphokinase increased | 1 (0.9) | 0 | 0 |
| Arthralgia | 1 (0.9) | 0 | 0 |
| Muscular weakness | 1 (0.9) | 1 (7.1) | 0 |
| Limb discomfort | 0 | 2 (14.3) | 0 |
| Hemorrhage subcutaneous | 1 (0.9) | 0 | 0 |
| Pain in extremity | 0 | 0 | 1 (16.7) |
| AESIs | | | |
| Subjects with at least one AESI, n (%) | 4 (3.4) | 1 (7.1) | 1 (16.7) |
| Muscular weakness | 1 (0.9) | 1 (7.1) | 0 |
| Constipation | 1 (0.9) | 0 | 0 |
| Dysphagia | 1 (0.9) | 0 | 0 |
| Urinary retention | 1 (0.9) | 0 | 0 |
| Dyspnea | 1 (0.9) | 0 | 0 |
| Cranial nerve paralysis | 0 | 0 | 1 (16.7) |
| Hemiparesis | 0 | 0 | 1 (16.7) |

AE, adverse event; AESI, adverse event of special interest; N, number of subjects in each group; n, number of subjects in the given category; OLEX, open-label extension period; SES, safety evaluation set.

**Fig. S1** Mean change in MAS-PF from baseline (Day 1) to Week 4 after each incobotulinumtoxinA injection in the OLEX (FAS)


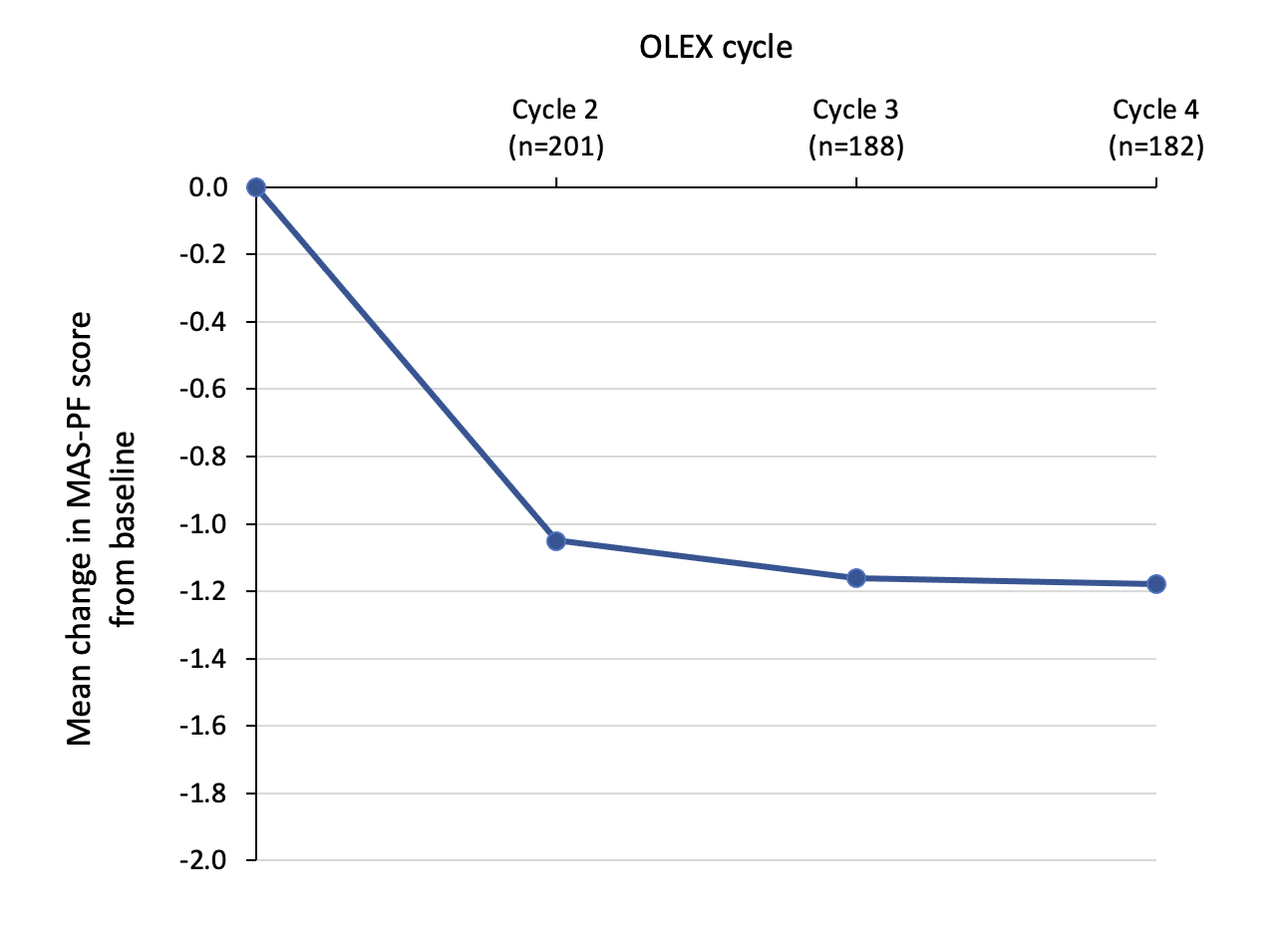
 FAS, full analysis set; MAS-PF, Modified Ashworth Scale spasticity score for the plantar flexors; OLEX, open-label extension period.

**Fig. S2** Change in MAS-PF from baseline (Day 1) to Week 4 after each incobotulinumtoxinA injection in the OLEX according to the length of the first and second OLEX injection cycles for patients with the same cycle length for these two injection cycles (FAS)


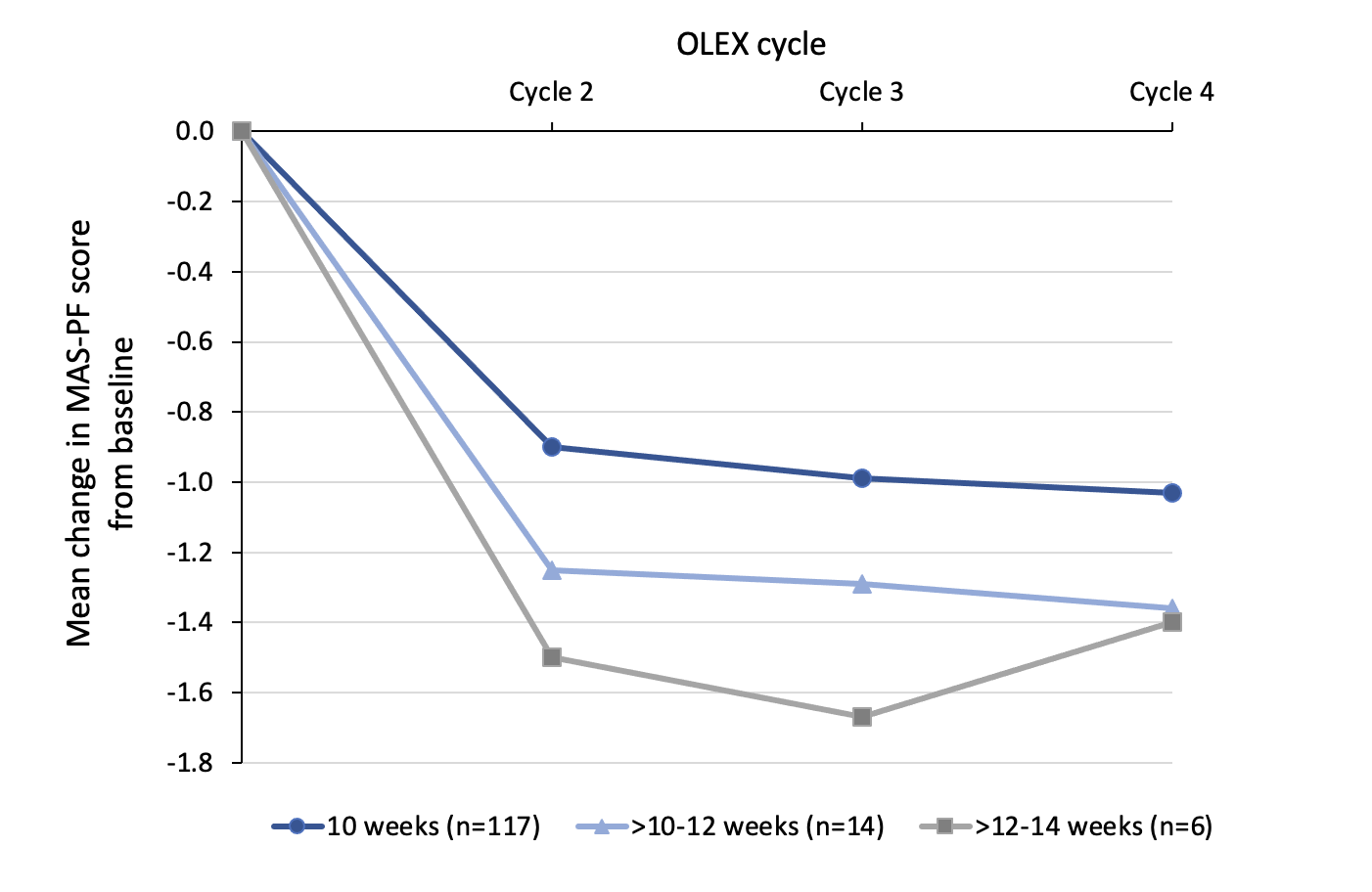


FAS, full analysis set; MAS-PF, Modified Ashworth Scale spasticity score for the plantar flexors; OLEX, open-label extension period.

**Fig. S3** Change in investigator’s CGI score from baseline (Day 1) to Weeks 4 and 12 in the MP, and to Week 4 after each incobotulinumtoxinA injection in the OLEX (FAS, BOCF for MP)


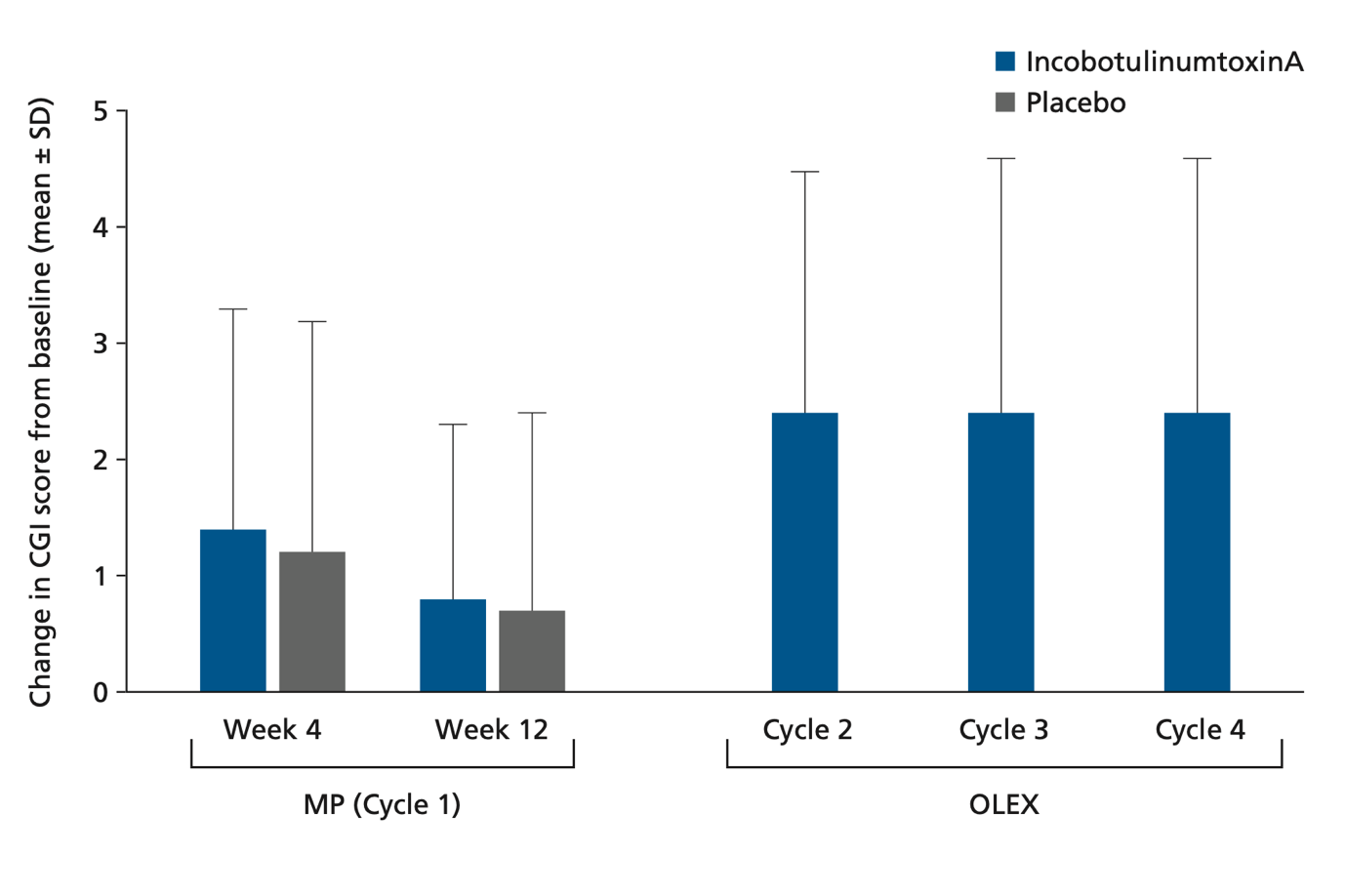


BOCF, baseline observation carried forward; CGI, clinical global impression; FAS, full analysis set; MP, main period; OLEX, open-label extension period; SD, standard deviation.

**Fig. S4** Change in total gait PRS score from baseline (Day 1) to Weeks 4, 8 and 12 in the MP (LS mean [SE]), and to Week 4 after each incobotulinumtoxinA injection in the OLEX (mean [SD]) (FAS, BOCF for MP)


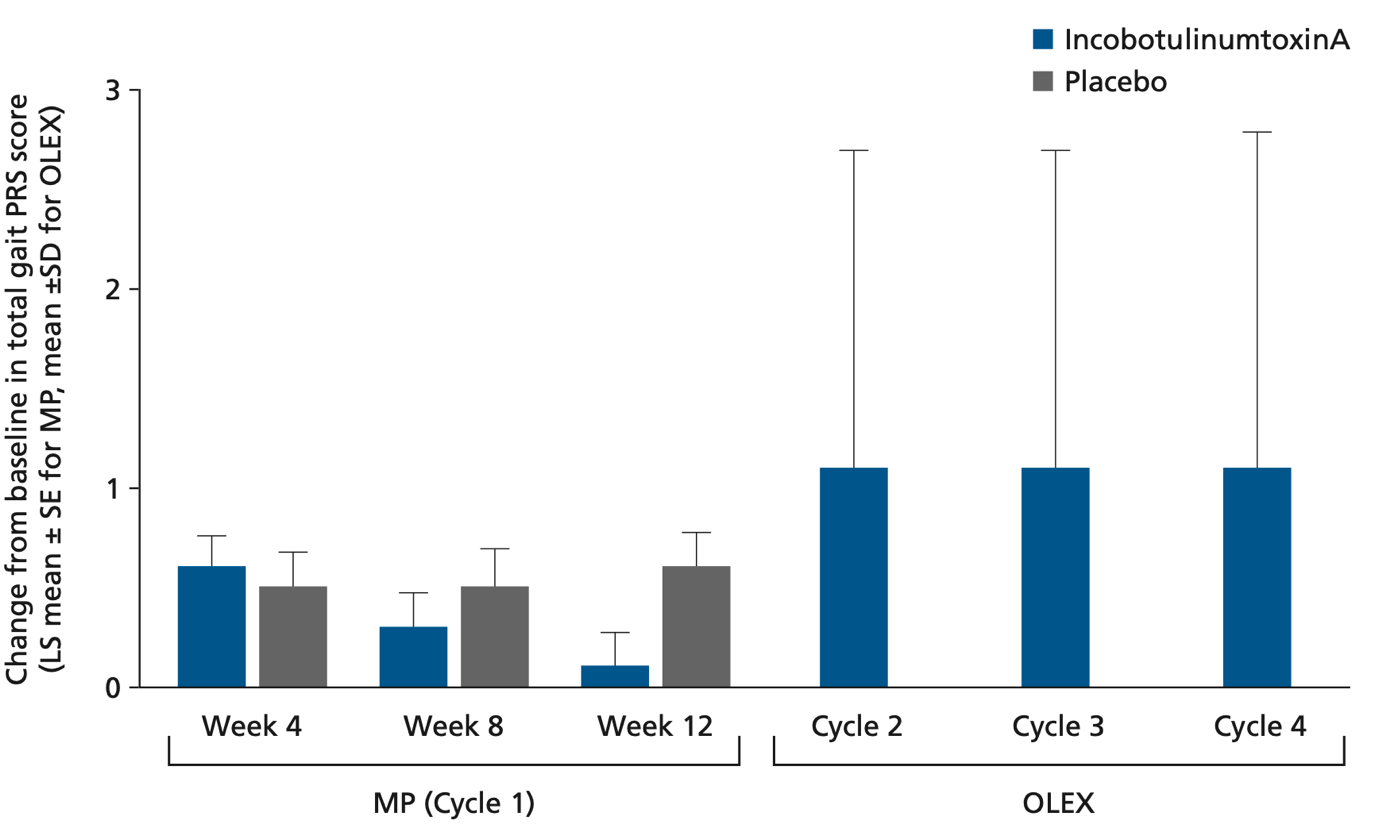


BOCF, baseline observation carried forward; FAS, full analysis set; LS, least squares; MP, main period; OLEX, open-label extension period; PRS, physician’s rating scale; SD, standard deviation; SE, standard error.
